# Genomic prediction and genome-wide association study for dagginess and host internal parasite resistance in New Zealand sheep

**DOI:** 10.1186/s12864-015-2148-2

**Published:** 2015-11-17

**Authors:** Natalie K. Pickering, Benoit Auvray, Ken G. Dodds, John C. McEwan

**Affiliations:** Invermay Agricultural Centre, AgResearch Limited, Puddle Alley, Private Bag 50034, Mosgiel, 9053 New Zealand; Focus Genetics Ltd, PO Box 12075, Ahuriri, Napier 4144 New Zealand; Department of Mathematics and Statistics, University of Otago, Dunedin, 9058 New Zealand

**Keywords:** Genome-wide association study, Sheep, Dagginess, Internal parasites

## Abstract

**Background:**

Dagginess (faecal soiling of the perineum region) and host nematode parasite resistance are important animal welfare traits in New Zealand sheep. Genomic prediction (GP) estimates the genetic merit, as a molecular breeding value (mBV), for each trait based on many SNPs. The additional information the mBV provides (as determined by its accuracy) has led to its incorporation into breeding schemes. Some GP methods give SNP effects, which provide additional information to identify genome-wide associations (GWAS) for a trait of interest. Here we report results from a GP and GWAS study for dagginess and host nematode parasite resistance in a New Zealand sheep industry resource.

**Results:**

Genomic prediction analysis was performed using 50K SNP chip data and parent average-removed, de-regressed BVs for five traits, from a resource of 8705 pedigree recorded animals. The five traits were dag score at three and eight months (DAG3, DAG8) and nematode faecal egg count in summer (FEC1), autumn (FEC2) and as an adult (AFEC). The resource consisted of Romney, Coopworth, Perendale, Texel and various breed crosses (designated: CompRCP, CompRCPT and CompCRP). The pure breeds, apart from Texel, plus CompRCP were used to develop the GP. The resulting SNP effects were used to identify genetic regions associated with dagginess and parasite resistance. Accuracies of the weighted correlation between mBV and true BV ranged between −0.07 (Texel) and 0.56 (Coopworth) for DAG3 and DAG8. For FEC1, FEC2 and AFEC accuracies ranged between −0.22 (CompRCPT) and 0.69 (Coopworth). The weighted average individual accuracy (calculated from theory) ranges were 0.13 (Texel) to 0.52 (Coopworth) and 0.11 (Texel) to 0.55 (Coopworth) respectively, for dagginess and parasite traits. There was one SNP for DAG8 that reached Bonferroni significance threshold (*P* < 1 × 10^−6^) on OAR15, the same two SNPs for each of the parasite traits (OAR26) and none for DAG3. A notable peak was also observed on OAR7 for all the parasite traits, however, it did not reach the Bonferroni significance threshold.

**Conclusions:**

This study presents the first results of a GWAS on dagginess and faecal egg count traits in New Zealand sheep. The results suggest that there are quantitative trait loci on OAR 15 for dagginess and on OAR26 and seven for faecal egg count.

**Electronic supplementary material:**

The online version of this article (doi:10.1186/s12864-015-2148-2) contains supplementary material, which is available to authorized users.

## Background

Dagginess (faecal soiling of the perineum region) and internal nematode parasites, are major animal health traits with ethical and welfare implications of interest to the New Zealand sheep industry. Firstly, dagginess has a known association with flystrike (genetic correlations range between 0.34 ± 0.13 and 0.81 ± 0.15) [1, 2], with the majority of flystrike occurring in the breech. Secondly, internal parasites are increasingly becoming resistant to anthelmintic drenches; there is known resistance to all major classes of anthelmintics [3]. Finally, there is a perception that internal parasite load is associated with level of dagginess. Breeding to reduce level of dagginess and internal parasite load as measured by faecal egg counts is a strategy that can provide cumulative and permanent progress.

Dagginess and parasite resistance, as measured by faecal egg counts of Strongyle (FEC) and *Nematodirus* egg count (NEM), have been shown to be moderately heritable traits [4], with estimates in New Zealand sheep of 0.37 and 0.34 for dag score at three and eight months (DAG3, DAG8) and between 0.18 and 0.21 for FEC/NEM traits [5]. Dagginess and FEC/NEM are often thought to be genetically associated in sheep. However, recent estimates showed that genetic and phenotypic correlations between FEC/NEM and dagginess traits (DAG3 and DAG8) were low to zero in New Zealand sheep [5]. A subsequent study also showed that dagginess was not correlated with wool length, bulk or type [6]. This does not rule out the immune response to worm burden irrespective to FEC/NEM as a cause of dagginess, but does suggest that the cause is internal and may involve processes within the intestinal tract.

Traditional genome-wide linkage studies which find quantitative trait loci (QTL) associated with a trait of interest has been used successfully in animal production [7, 8]. The sequencing of many domesticated species: e.g. cattle, chicken and sheep, have allowed the introduction of high-density SNP genotyping platforms. These involve thousands and for some species hundreds of thousands of SNPs approximately equally spaced across the genome, to capture the greatest amount of linkage disequilibrium with causative QTL. This has produced rapid progress in genome-wide association studies (GWAS) which have already identified regions associated with production [9], fertility [10], disease [11–14] and polledness [15] traits in cattle and sheep.

The same platforms have given rise to marker assisted selection on a genome-wide scale, called genomic prediction or selection [16]. The sum of the effect each SNP has on a trait is used to predict the animals’ molecular breeding values (mBVs) [17]. Thus potentially all the genetic variation for a trait could be picked up by the SNP panel due to the extent of LD between the SNPs on the panel and causative QTL. The dairy industry has already adopted GP to increase genetic gain [18], and it has been recently implemented in the New Zealand sheep industry [19].

A resource consisting of greater than 3.5M pedigree recorded animals, born between 1990 and 2010 from 233 industry recorded flocks, with estimated breeding values (eBVs) for a number of production traits, including DAG3, DAG8 and FEC in summer (FEC1), autumn (FEC2) and as adult (AFEC), was available for use. Of these, 8705 have been genotyped on the Illumina Ovine SNP50BeadChip (50K). The aim of this study was to estimate the accuracy of mBVs for these traits, using genomic BLUP, which assumes all SNPs have a small effect and are normally distributed. A second aim was to use the SNP effects generated from the genomic prediction analysis to identify regions associated with these traits, in a GWAS.

## Results and discussion

### Quality control

A step by step quality control pipeline was performed [20]. From the initial set of 54,977 useable SNPs, 4869 were not retained by the Ovine HapMap [21] and a further 1781 SNPs were discarded due to one or more of the following; non-autosomal (including pseudoautosomal), minor allele frequency (MAF) = 0, call frequency <0.97 and Illumina quality score (GC10) value <0.422. The final dataset included 8705 animals and 48,327 SNPs.

### Summary of dependent variables

Of the 3.5M animals used for eBV estimation there were 95,544 and 75,979 raw measurements for DAG3 and DAG8, respectively. The traits FEC1 (scored in summer) and FEC2 (scored in autumn) are repeatable traits with two samples (a and b) potentially collected at each time point, several days apart (Table 1). For AFEC, this trait is not recorded and the eBVs are generated using estimated genetic and phenotypic correlations with other traits including FEC1 and FEC2. Table 1 summarizes the raw measurements used by Sheep Improvement Limited (SIL), the New Zealand sheep genetic evaluation database, to generate the eBVs and the resulting dependent variables (*y*) used for molecular breeding value (mBV) calculation; i.e. are parent averaged de-regressed, have reliabilities greater or equal to 0.8 times the heritability and were for animals genotyped on the 50K SNP chip. There were between 1957 and 4164 animals for each trait with *y* values; corresponding reliabilities were between 0.34 and 0.51.

**Table 1:** Summary of raw phenotypes, de-regressed dependent variables with parent average removed and reliabilities

	Phenotypes				*y*			rel	
Trait	n	Mean	sd	h^2^	n	Mean	sd	Mean	sd
DAG3	95544	0.93	1.26	0.33	2640	−0.03	1.03	0.47	0.33
DAG8	75979	1.23	1.45	0.31	1957	−0.13	1.08	0.51	0.31
FEC1a	124948	1020.32	1418.94	0.16	4164	−0.17	0.64	0.42	0.21
FEC1b	37976	999.20	1180.52						
FEC2a	105215	1194.57	1548.24	0.20	3269	−0.16	0.75	0.34	0.20
FEC2b	49289	1177.50	1409.65						
AFEC	0			0.25	2204	−0.20	0.84	0.35	0.25

*n* number, *sd* standard deviation, *h* ^*2*^ heritability, *y* de-regressed dependent variables with parent average removed (*y*), *rel* reliabilities of *y*, *DAG3, DAG8* dag score at three and eight months, respectively, *FEC1, FEC2, AFEC* nematode faecal egg count in summer, autumn and as an adult, respectively (a and b = repeat measures)

The dependent variables were split into a training and validation datasets, based on birth year, for the genomic prediction and to estimate accuracy of the prediction equations. This was performed for each breed (Romney, Coopworth, Perendale, Texel and three breed crosses designated: CompRCP, CompRCPT and CompCRP) and trait (Table 2). Training set cut offs were chosen to ensure adequate numbers were in the training and validation datasets, see methods for a complete description.

**Table 2:** The year of birth of the first animals placed in the validation set and number (n) of animals in training and validation sets for each breed

	First validation year				n Training				n Validation						
Trait	R	C	P	RCP	R	C	P	RCP	R	C	P	RCP	T	RCPT	CRP
DAG3	2008	2009	2004	2009	624	622	52	188	221	234	56	276	86	158	123
DAG8	2008	2005	2004	2009	715	209	50	72	278	245	53	83	86	85	81
FEC1	2008	2009	2005	2008	1414	1033	164	222	264	239	185	204	124	160	155
FEC2	2008	2009	2005	2007	1168	917	175	101	165	95	193	137	98	97	123
AFEC	2006	2005	2004	2005	771	381	123	10	252	237	170	66	76	54	64

*Rom* Romney, *Coop* Coopworth, *Peren* Perendale, *RCP* CompRCP, *RCPT* CompRCPT, *CRP* CompCRP, *PC* principal components, *DAG3, DAG8* dag score at three and eight months, respectively, *FEC1, FEC2, AFEC* nematode faecal egg count in summer, autumn and as an adult, respectively

### Principal component analysis

**Fig. 1:**
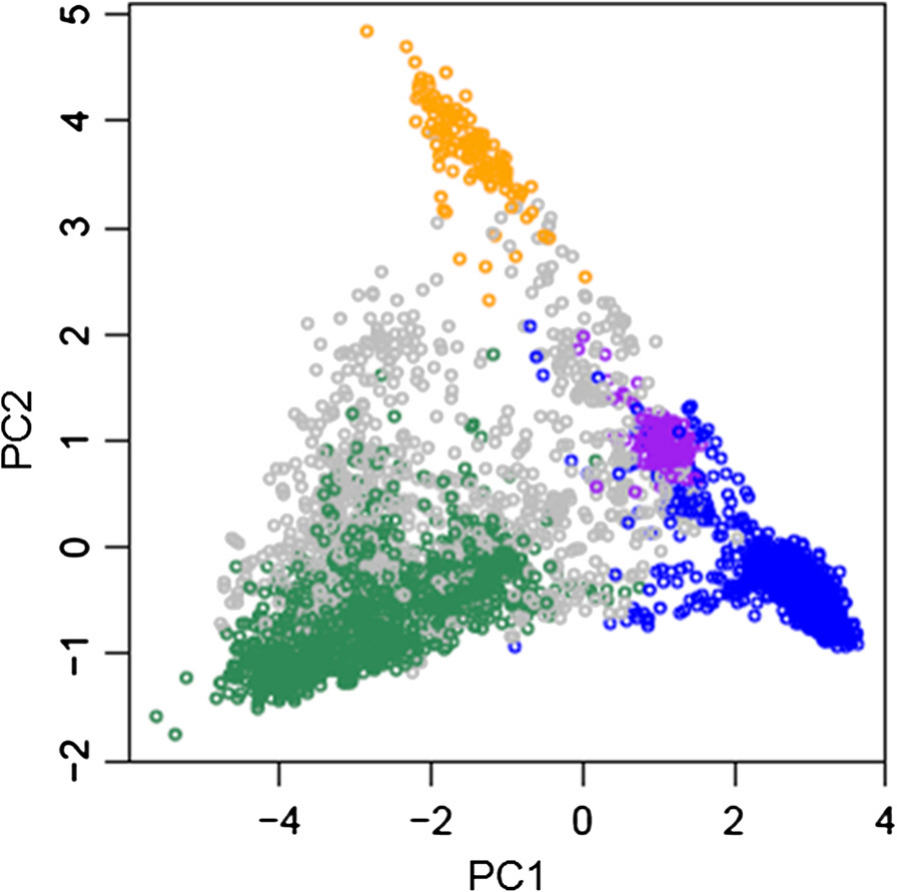
The first two principal components (PC) calculated for all animals for faecal egg count in summer. Romney (blue), Coopworth (green), Texel (yellow), Perendale (purple) and others/composites (grey)

Using the G1 matrix, described by VanRaden [22], the first six principal components (PC) using the animals in the training set were calculated. The six PCs accounted for between 0.60 and 0.73 of the genetic variation contained in the genomic relationship matrix for each trait (Table 3). In Fig. 1, the first (PC1) and second (PC2) largest axes of variation are plotted using the animals with FEC1 *y* values. The distinction of the four main breed groups (Romney, Coopworth, Perendale and Texel), with the three composites breeds (shown as ‘other’) clustered in-between, are shown clearly and is typical of the New Zealand sheep industry.

**Table 3:** The genetic variance explained by the first 6 principal components for each trait

Trait	PC1	PC2	PC3	PC4	PC5	PC6	Total
DAG3	0.57	0.06	0.03	0.02	0.02	0.01	0.71
DAG8	0.44	0.06	0.03	0.03	0.02	0.02	0.60
FEC1	0.59	0.04	0.04	0.02	0.02	0.02	0.73
FEC2	0.57	0.05	0.03	0.03	0.02	0.02	0.71
AFEC	0.44	0.06	0.04	0.04	0.02	0.01	0.60

*PC* principal components, *DAG3, DAG8* dag score at three and eight months, respectively, *FEC1, FEC2, AFEC* nematode faecal egg count in summer, autumn and as an adult, respectively

### Accuracy of genomic prediction

The accuracies of the five traits calculated as the adjusted weighted correlation between mBV and *y* (r_A_) and as the weighted average individual accuracy (r_I_) are shown in Table 4. These were calculated using the animals in the validation set. For the dagginess traits the r_A_ ranged between 0.11 and 0.56 for those breeds in the training set and r_I_ ranged between 0.31 and 0.52. The Perendales had the lowest r_A_ and r_I_ reflecting the low number of animals from this breed in the training set; n = 52 and 50 for DAG3 and DAG8, respectively (see methods). For the three breeds present in the validation set only (Texel, CompRCPT and CompCRP) r_A_ and r_I_ were poor for the Texel who are the furthest removed from the validation set. The two composites have more than 30 % of their genetic background from Romney, Coopworth or Perendale breeds and had accuracies close to values seen by the breeds represented in the training set.

**Table 4:** Accuracies as the weighted correlation between mBV and dependent variable (r_A_) and the weighted average individual accuracy (r_I_) calculated for the five traits in the seven validation breeds

	Rom		Coop		Peren		RCP		Texel		RCPT		CRP	
Trait	r_A_	r_I_	r_A_	r_I_	r_A_	r_I_	r_A_	r_I_	r_A_	r_I_	r_A_	r_I_	r_A_	r_I_
DAG3	0.34	0.46	0.56	0.52	0.26	0.35	0.41	0.44	−0.07	0.16	0.35	0.42	0.39	0.40
DAG8	0.40	0.47	0.41	0.43	0.11	0.31	0.31	0.35	0.15	0.13	0.40	0.33	0.16	0.30
FEC1	0.40	0.51	0.71	0.55	0.22	0.41	0.65	0.49	0.03	0.21	0.39	0.51	0.50	0.46
FEC2	0.49	0.51	0.69	0.49	0.18	0.39	0.68	0.46	0.09	0.18	0.26	0.41	0.66	0.34
AFEC	0.27	0.35	0.24	0.35	0.24	0.28	0.29	0.33	0.10	0.11	−0.22	0.28	0.33	0.23

*Rom* Romney, *Coop* Coopworth, *Peren* Perendale, *RCP* CompRCP, *RCPT* CompRCPT, *CRP* CompCRP, *r*_*A*_ weighted correlation between mBV and dependent variable, *r*_*I*_ weighted average individual accuracy, *PC* principal components, *DAG3, DAG8* dag score at three and eight months, respectively, *FEC1, FEC2, AFEC* nematode faecal egg count in summer, autumn and as an adult, respectively

For the FEC traits, the r_A_ ranged between 0.18 and 0.71 for those breeds represented in the training set and between 0.28 and 0.55 for r_I_. The Perendales again had the lowest accuracies; the number of animals of this breed in the training set were low (n = 164, 175 and 123, for FEC1, FEC2 and AFEC, respectively). The composite (CompRCP) also had a low number of animals in the training set for FEC2 and AFEC, 101 and 10, respectively. The slightly higher accuracies seen for CompRCP compared to the Perendales may be due to the CompRCP animals consisting of at least 50 % Romney, Coopworth and/or Perendale. Again, of the breeds represented only in the validation sets, the Texel had the lowest r_A_ and r_I_ except for AFEC, where CompRCPT had a r_A_ of −0.22. Adult FEC had lower accuracies for most breeds compared to FEC1 and FEC2. This is probably a reflection of the lower numbers of animals available with *y* values for this trait, which in turn is due to this trait being indirectly predicted from correlated traits.

The accuracies were compared to the theoretical accuracies using equation 8 from Goddard [23], following the assumptions of an effective population size (Ne) of 405 (New Zealand Romney, Table S4 [21]), number of records available per trait and genome length of 30M. The theoretical accuracies were 0.18 and 0.20 for DAG3 and DAG8 respectively and 0.32, 0.31 and 0.23 for FEC1, FEC2 and AFEC, respectively. The accuracy estimates obtained in this study are higher than those calculated theoretically. The reason for the higher accuracies is that the theoretical values are for ‘unrelated’ animals i.e. ~10 generations or more distant. In practice, most of the validation animals have an ancestor 1–3 generations distant in the training data set and as such will have higher estimated accuracies than expected from theory.

Simulations showed that when there are limited numbers of animals from one population set, then the most accurate genomic predictions are generated when information from all populations are combined in the training set rather than predicting separately by population [24]. However, the more genetically diverse the populations are, the less accurate are the genomic predictions for across breed analysis. This corroborates the low accuracy for the Texels in this analysis, as they are the most divergent breed in the validation set compared to those present in the training set. The Texels originated from Texel an island offshore from the Netherlands, while the Romneys were from England, their estimated divergence is 160 to 240 generations ago [21]. To increase accuracies for Texels, more animals are required so that some may be combined in the training set. This may in part be achieved by increasing the number of composites with at least 50 % Texel, if pure-breds are hard to collect.

The accuracies for the CompRCPT and CompCRP are higher than expected for a breed not present in the training set, however, they are at least 50 % and at least 30–50 %, respectively, of the breeds represented in the training set. The strength of the genetic relationships between individuals and breeds was shown in the principal component analysis, e.g. for FEC1 (Fig. 1).

Implementation in industry in New Zealand for these traits currently uses the mBVs as described here with minor modifications [25]. These are then blended with eBVs calculated on all available animals (see Dodds [26] for a brief description).

### GWAS

**Fig. 2:**
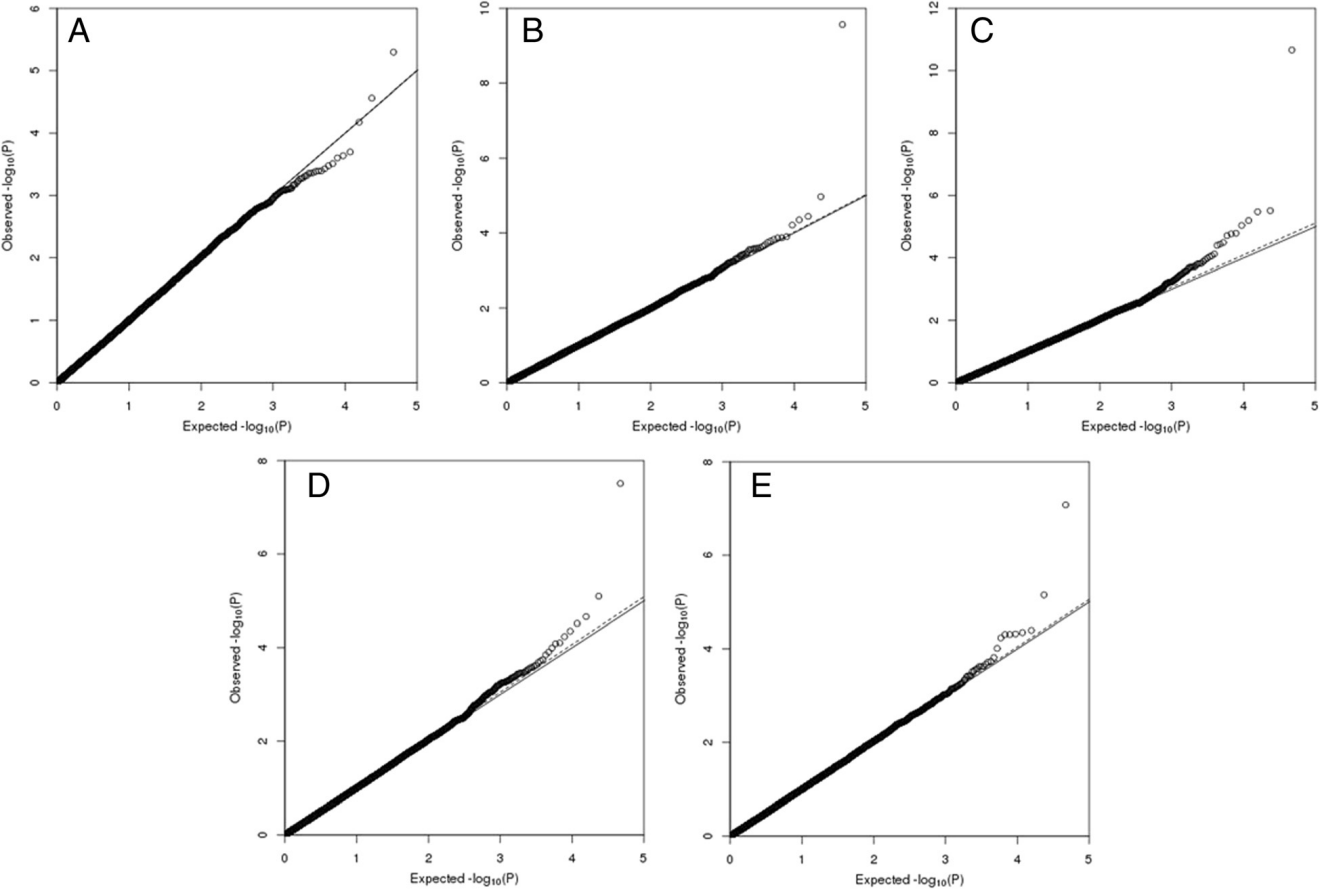
Quantile-quantile plot for dag score at three (**a**) and eight months (**b**), faecal egg count in summer (**c**), autumn (**d**) and as adult (**e**). The 0–1 line (solid) and the slope (dash) are also plotted

The quantile-quantile (Q-Q) plots (Fig. 2) showed that the deviation of the majority of observed -log_10_(*P*) values from the expected values was insignificant (lambda ranged between 1.001 and 1.021). The SNPs seen to be deviating from the expected values were interpreted as SNPs associated with the trait of interest, as the SNPs are departing from the null hypothesis of no genetic association and no LD between SNPs. There were 32 regions associated with DAG3, DAG8, FEC1, FEC2 and/or AFEC with a P value < 0.0001 (Additional file 1).

**Fig. 3:**
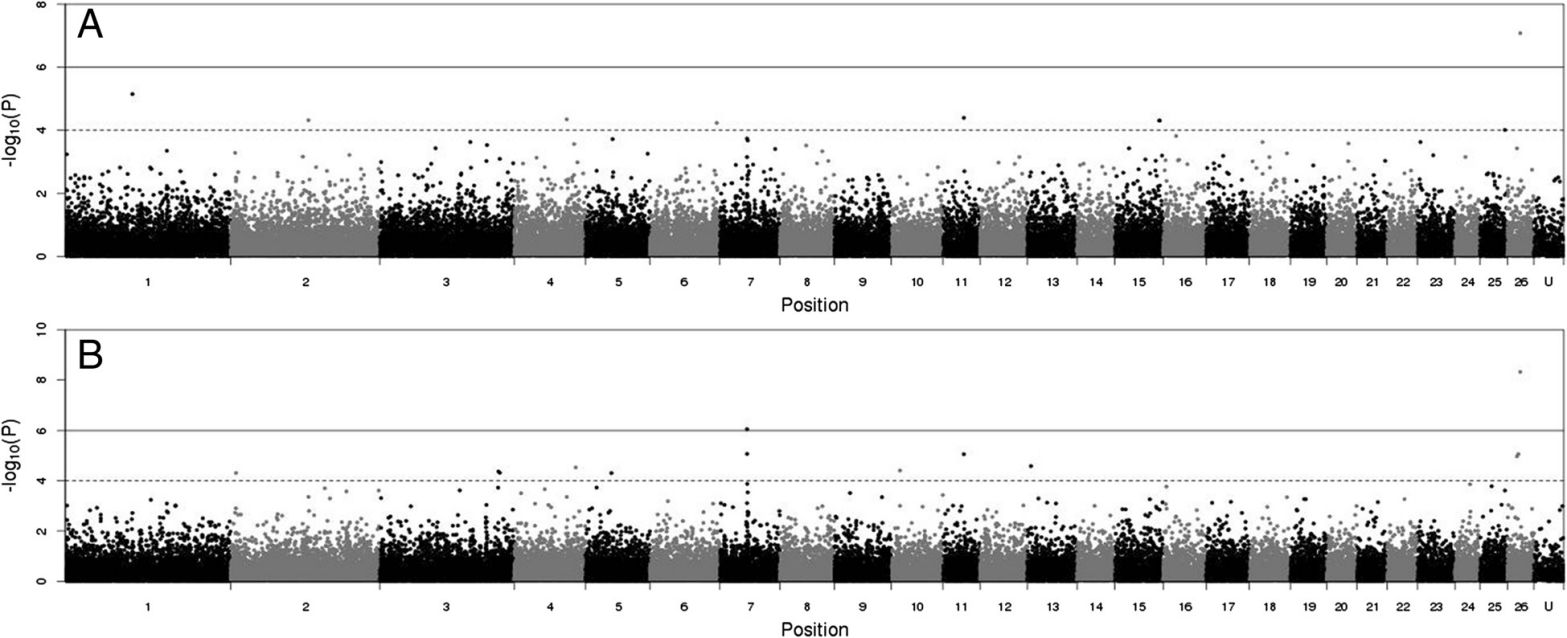
Manhatten plot of -log_10_(*P*) values of SNPs for dag score at eight months (**a**) and faecal egg count in summer (**b**). Ordered on the ovine genome v3 map, *P* < 0.0001 (solid line), *P* < 0.001 (dash line)

**Fig. 4:**
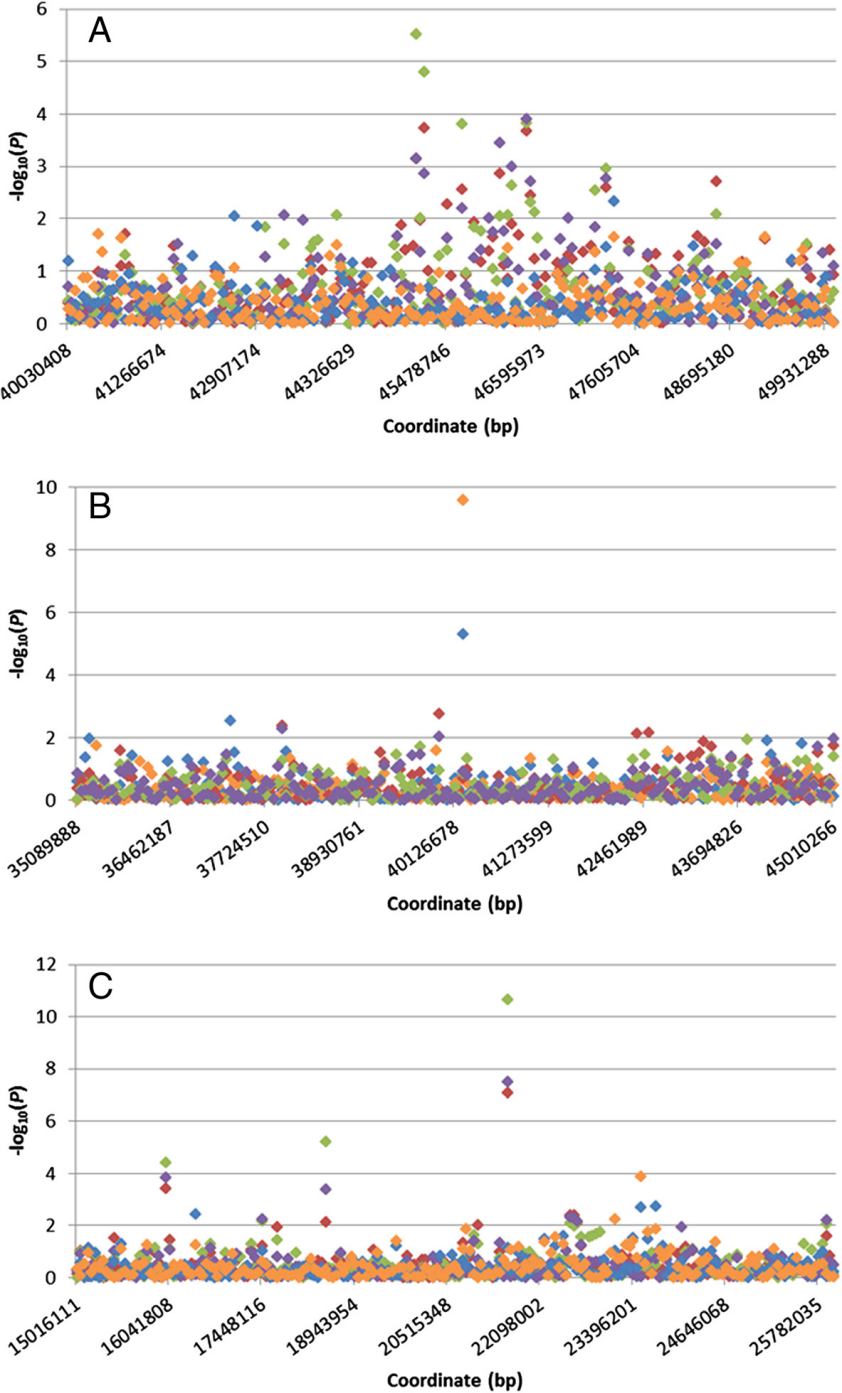
Distribution of -log_10_(*P*) values for dag score at three (blue) and eight months (orange) and faecal egg count in summer (green), autumn (purple) and as adult (red) for candidate regions on OAR 7 (**a**), 15 (**b**) and 26 (**c**)

Figure 3 (a and b) show the Manhattan plots of the resulting -log_10_(*P*) values for DAG8 and FEC1, respectively. The Manhattan plot for DAG3 was similar to DAG8, and plots for FEC2 and AFEC were similar to FEC1 (Additional file 2). A summary of genes underlying the top SNPs with a P value < 0.0001 for each trait is in Additional file 1. For DAG3 and DAG8 there was one peak common to both traits detected on OAR15 (Fig. 4b), comprising of a single SNP (s22390; *P* value 5.04 × 10^−6^ and 2.72 × 10^−10^, respectively). Annotation on Ovine genome v3.1 (http://www.ensembl.org/Ovis_aries) showed there are no known genes or proteins within 100kbp window of this SNP. Two predicted genes were observed (Ensembl transcript: GENSCAN00000038546 and GENSCAN00000038543), however RNA-seq data at Ensembl does not provide supporting evidence for these genes being real. The 100kbp sequence was scanned for open reading frames in all 6 frames, these were matched against a collection of protein signature databases using InterProScan 5 [27]. This identified three matching domains; integrin beta subunit, insulin-like growth factor binding protein and Agouti (Additional file 3).

For the FEC traits there were two distinguishable similarities on OAR 7 and 26 (Fig. 4a and c). On OAR 7 there was a notable peak consisting of 17 SNPs in total for all three traits, spanning a 3.36 Mbp region (45,194,749bp to 48,549,329bp), with the top SNP within this region passing the *P* < 10^−4^ threshold for FEC1 (s65809). On OAR 26 there was one significant peak (OAR26_25273391), with a *P* value ranging between 8.38 × 10^−8^ and 2.18 × 10^−11^ for FEC1, FEC2 and AFEC.

The gene positioned under the peak on OAR 7 is the vacuolar protein sorting 13 homolog C (VPS13C; OMIM: 608879). This family of proteins are involved in the trafficking of membrane proteins between the *trans*-Golgi network and the prevacuolar compartment (*Saccharomyces cerevisiae*) corresponding to the multivesicular body/late endosome in mammals [28]. Four splice variants of this gene have been observed, along with three repeat regions. Homolog C arose from duplication of homolog A, also known as chorein, which is involved in chorea-acanthocytosis (ChAc), an autosomal recessive disease. However, homolog C cannot compensate for defunct homolog A in ChAc patients [28]. The VSP13 family of proteins are expressed in most tissues including the small intestine and colon [28].

The gene positioned under the peak on OAR 26 is the zeta-sarcoglycan (SGCZ; OMIM: 608113) gene. This gene is involved in the formation of the sarcoglycan (SG) complex with SGCE, SGCB and SGCD in smooth muscle, retina and Schwann cells [29, 30]. The SG complex is part of the dystrophin-glycoprotein complex that interacts between the actin cytoskeleton and the extracellular matrix, essential for membrane stability. Mutations in the sarcoglycans cause limb-girdle muscular dystrophy, with malfunctions of digestive smooth muscle leading to dysphagia, vomiting, chronic constipation and acute digestive dilatations. This region also overlaps with a copy number variant (CNV) region associated with obesity in mice [31].

Numerous studies have been carried out to investigate the genetic control of resistance to internal parasites. The majority of these studies involved microsatellite-based linkage studies [32–38]. There are only a few more recent studies involving SNP chip data [11, 12, 39, 40], one published study using both microsatellites and SNPs [41] and one using candidate gene approach [42]. Numerous traits representing parasite resistance have been used in these previous studies, for example, immunoglobulin A activity, packed cell volume and eosinophil counts, as well as the standard FEC and NEM traits. Given this, of the 32 identified SNPs with a *P* < 0.0001, there were 16 regions which overlapped previous QTL/GWAS studies on gastrointestinal parasites (Additional file 4). Notably the region on OAR 7 (~45.3cM) overlapped with four other studies [12, 36, 40, 41]. A region on OAR 2 (~129.9cM) also overlapped or is near regions identified in five other studies [11, 32, 33, 36, 39]. Four regions associated with DAG3 and/or DAG8 in this study were also identified in previous studies on gastrointestinal parasites (OAR 1 ~ 154.6cM [11, 35]; OAR 3 ~ 138.4cM; [33]; OAR 8 ~ 71.1cM [39]; and OAR 15 ~ 40.2; [11, 38, 41]). It has been reported in a comprehensive dataset that FEC and dag score traits are not strongly genetically or phenotypically correlated, however, it was suggested that parasite worm burden could still play a role in faeces accumulation [5]. Even though no similarities between significant regions were found between the two trait sets in this study, there may be similarities in the genes involved in the whole physiological response. It could be that the part of the response involved in reducing FEC/pasture contamination is a different gene set from those involved in what leads to faecal accumulation. Therefore, similarities between regions associated with dag score in this study and FEC from previous studies could arise, relating to the physiological response to the parasitic infection.

## Conclusions

These results indicate that genomic prediction can be implemented for most breeds in the New Zealand sheep industry for dagginess and FEC traits. In addition, three regions have been identified, one on OAR15 shows association with faecal accumulation and two regions (OAR7 and OAR26) show an association with the FEC traits. This study adds additional information in the quest for understanding the genes potentially involved in the host response to internal parasites and faecal accumulation.

## Methods

This study was carried out in strict accordance of the guidelines of the 1999 New Zealand Animal Welfare Act and was approved by the AgResearch’s Invermay Animal Ethics committee.

### Estimated breeding values and dependent variables

Estimated breeding values (eBVs) were available for DAG3, DAG8, FEC1, FEC2 and AFEC from analyses performed by Sheep improvement Limited (SIL), the New Zealand sheep genetic evaluation system [43]. The eBVs were generated from approximately 3.5M pedigree recorded animals from 230 industry recorded flocks. To ensure SNP associations were not due to pedigree information, dependent variables (*y*) were calculated taking into account the individuals own and descendants’ information. Parent average effects are removed [44], assuming all genetic variation is explained by the markers (c = 0). The resulting values were de-regressed using the reliability of the eBV with parent-average removed.

The eBVs were available directly from SIL, however, for completeness the following is a description of the measurement criteria of the traits for input into the SIL database. Dagginess is a subjectively, visually-assessed trait scored at approximately three and eight months of age (DAG3; DAG8) using a 6-point scale: zero (no dagginess) to five (complete coverage of the breech and down the legs by faecal material) [6]. Measurements on FEC traits for input into SIL for BV estimation are done through the WormFEC™ programme [45]. Faecal egg count is a repeatable trait with BVs estimated from two samples (taken several days apart) taken in summer (FEC1) and autumn (FEC2). Egg counts are made of Strongyle (primarly *Ostertagia* spp, *Trichostrongylus* spp, *Cooperia curticei,* and in a proportion of farms *Haemonchus contortus* [46]). Breeding values for AFEC are estimated from genetic and phenotypic correlations with FEC1 and FEC2.

### Genotypes and quality assurance

Of the 3.5M pedigree recorded animals, 8705 had been genotyped, and passed quality control, with the Illumina® Ovine 50K SNP chip, according to the manufacturer’s protocol. These were mainly sires, only 22 % were female, and were predominately Romney, Coopworth, Perendale or Texel, plus other breeds and various crosses and composites. Genotyping results were put through a quality control pipeline before analysis [20]. In summary, SNPs are discarded if they have a call rate <97 %, appear non-autosomal (including pseudoautosomal), minor allele frequency (MAF) ≤0.01, Illumina quality score (GC10) value <0.422 and departed from Hardy Weinberg disequilibrium (1 × 10^−6^). The SNPs that were not retained as part of the Ovine HapMap study [21] were also discarded.

### Genomic prediction analysis

#### Breed designation and reliability threshold

Data were filtered on breed and reliability before analysis. Breed was designated by the following conditions: Romney, Coopworth and Perendale were reported if their breed composition was greater or equal to 75 %. There were also three composite breeds considered, based on the breed composition of the New Zealand sheep industry. Firstly, CompRCPT were those that were greater than 50 % of combined Romney, Coopworth, Perendale breeds and at least 25 % Texel. Secondly, CompRCP were those that were greater than 50 % of combined Romney, Coopworth, Perendale breeds and less than 25 % Texel. Finally CompCRP were those that had greater than 30 % and less than or equal to 50 % of combined Romney, Coopworth and Perendale breeds.

The reliability cut off was 80% of the heritability estimate used for eBV estimation. Animals had to have dependent variable reliabilities equal to or above this cut off to be considered for analysis as typically they have either not been measured or alternatively progeny tested for the trait. The number of animals in the final analysis (i.e. with both genotypes and eBVs above cut off) were 2640 for DAG3 (44 % female), 1957 for DAG8 (31 % female), 4165 for FEC1 (33 % female), 3269 for FEC2 (27 % female) and 2204 for AFEC (16 % female).

### Training and validation assignment

After the above filtering, genotypes were scored on the number of copies of the ‘A’ allele (based on Illumina AB calling format). Missing genotypes were filled in using the breed mean, estimated using a least squares regression on breed proportions as Romney, Coopworth, Perendale, Texel and other, to generate allele frequencies for each SNP within breed. The missing values are then replaced weighted by the individuals breed proportion of Romney, Coopworth, Perendale, Texel or other.

Training and validation sets were formed to a) derive a prediction equation using the training set and b) to estimate the accuracy of the prediction equation in the validation set. For estimating the SNP effects for the GWAS all animals were used in the training set. The animals were split into validation and training sets based on birth year (Table 2). The Texel, CompRCPT and CompCRP animals were only used in the validation set, to see how well the predictions work for these groups when not directly in the training set, as well as for groups represented in training. Training set cut off years were chosen for each breed, using a number of criteria. First, at least 200 animals per breed are used for validation. Secondly, if there are less than 400 animals roughly half are required in each set. Finally, if there were between 75 and 100 animals then a small portion (~10) were left in the training set, and the rest in validation.

The dataset comprised mainly of males used as sires, for each trait the percentage of females in the training and validation sets were: 39 and 50 % for DAG3, 20 and 43 % for DAG8, 30 and 38 % for FEC1, 26 and 32 % for FEC2 and 9 and 27 % for AFEC, respectively.

### GBLUP

For full description of methods see [23], in summary the following methods were applied. Two genomic relationship matrices were used. The first G matrix (**G1**), as described by VanRaden [22] was used to calculate the coefficients (i.e., a linear prediction equation), while the second G matrix (**G2**, calculated using breed-specific allele frequencies [47]) was used to calculate the individual accuracies as described below. In a multi-breed population the G2 matrix is more similar to the pedigree-derived relationship matrix than G1, [47] and [23] recommend using G2 in preference to G1 for calculating individual accuracies.

The mBVs were calculated using genomic BLUP method of VanRaden [22]. A mixed model was fitted to the dependent variable, ***y***, for each trait as follows: ***y*** = **Xβ** + **Z*u*** + **e** where **X** is a matrix of the first six principal components of the **G1** [22] matrix (to account for population stratification), **β** is a vector of fixed effects of the PC, **Z** is an incidence matrix and ***u*** is the animal effects (breeding values) distributed as N(0, **G1** σ^2^_u_), where σ^2^_u_ is the additive genetic variance and **e** are the residual effects distributed as N(0,**R**) where **R** is a diagonal matrix with diagonal elements (1-r^2^)/r^2^ where r^2^ is the reliability of ***y***.

The mBVs are the predicted animal effects from the above model. The mBVs were obtained by multiplying the SNP effects by the SNP genotypes and summing.

### Calculating the accuracy

The accuracies of the mBVs for each breed were derived from the validation animals using two different methods. The first method used the mBVs from GBLUP fitting the G1 matrix; ${r}_A = \frac{cor\left(\boldsymbol{y},\boldsymbol{M}\boldsymbol{B}\boldsymbol{V}\right)}{h_g}$, and was weighted by 1/(1-r^2^). The mBV were calculated as above, only using the training set. The effective heritability (h^2^_g_) is equal to the average reliability (r^2^) of ***y***. The second method uses the prediction error variance (PEV) [48] from a genomic BLUP analysis fitting the **G2** matrix, giving; ${r}_{Ii}=\sqrt{1-\frac{PE{V}_i}{\sigma_u^2}}$ for animal *i*, where σ^2^_u_ is the genetic variance and the PEV_*i*_ are obtained by inverting the left hand side of the mixed model equation [47]. These were calculated for all validation animals and averaged (weighted by 1/(1-r^2^)) to give an accuracy, *r*_*I*_*,* for each breed.

### GWAS

To identify genetic regions associated with the five traits, SNP effects (*b*_*i*_) were obtained from the above genomic BLUP using the G1 matrix and all animals in one dataset. Probability (*P*) values were calculated for the i^th^ SNP assuming the *b*_*i*_ follow a normal distribution with mean zero and variance: $var=\frac{2 pi\left(1- pi\right){n}_b{\sigma}_b^2}{{\displaystyle \sum \left(2 pi\left(1- pi\right)\right)}}$, where p_*i*_ is the frequency of the A allele of the *i*^th^ SNP in the population, σ^2^_b_ is equal to the empirical variance of b_*i*_ and n_*b*_ is the number of SNPs with effects. The -log_10_(*P*) values corresponding to the estimates of the *b*_*i*_ were graphed in a Manhattan plot on Ovine genome v3.1 [49] (available at Ensembl http://www.ensembl.org/Ovis_aries), and thresholds set at an initial level calculated using the 5 % Bonferroni correction [50] 0.05/n_*b*_ ≈ 10^−6^ (−log_10_(*P*) ~6).

The quantiles were calculated to check whether the distribution of the observed -log_10_(*P*) values deviated from the expected distribution (exponential) under the null hypothesis of no genetic association and no LD between SNPs. To do so, the n_*b*_ -log_10_(*P*) values were sorted and plotted against the -log_10_(1-u) where u = [1 / (n_*b*_ + 1), 2 / (n_*b*_ + 1), …, n_*b*_ / (n_*b*_ + 1)] as a quantile - quantile (QQ) plot.

### Exploration of significant SNPs

For peaks that reached the Bonferroni threshold, the genomic region was explored by inspecting a 100kbp window surrounding the location of the significant SNP using ovine genome v 3.1 (available at Ensembl http://www.ensembl.org/Ovis_aries). A further literature search and Online Mendelian Inheritance in Man (OMIM) were used to identify candidate genes.

### Supporting material

The data sets supporting the results of this article are included within the article and its additional files.

## Additional files

Additional file 1:
**List of genes within 100kbp region surrounding the best SNPs with -log**_**10**_ **(** ***P*** **) > 4 for each trait, listed by OAR (chr) and coordinate on Ovine genome v3.1.** (XLSX 11 kb) Additional file 2:
**Manhatten plot of -log**_**10**_ **(** ***P*** **) values of SNPs for dag score at three months (A) and faecal egg count in autumn (B) and as adult (C).** Ordered on the ovine genome v3 map, *P* < 0.0001 (solid line), *P* < 0.001 (dash line). (PNG 916 kb) Additional file 3:
**Output of protein domain matches from InterProScan 5 search of the 100kbp window around SNP s22390 located at OAR15: 40210579 on OARv3.1.** (CSV 110 kb) Additional file 4:
**Summary of quantitative trait loci for resistance to internal parasite traits from published papers, related to regions identified within the current study.** Positioned on ovine genome map v3, including chromosome (chr) position (Coordinate, cM), test statistic and significance and quantitative trait loci (SNP or MicroSatellite). (XLSX 13 kb)

## Abbreviations

GWAS: Genome-wide association study
PC: Principal component
LD: Linkage disequilibrium
SNP: Single nucleotide polymorphism
M: Million
MAF: Minor allele frequency
QTL: Quantitative trait loci
eBVs: Estimated breeding values
gBVs: Genomic breeding values
DAG3: Dag score at three months
DAG8: Dag score at eight months
FEC1: Faecal egg count in summer
FEC2: Faecal egg count in autumn
AFEC: Adult faecal egg count
QQ: Quantile-quantile plots
PEV: Prediction error variance
