# Supplementary figures and images for: Genomic prediction and genome-wide association study for dagginess and host internal parasite resistance in New Zealand sheep
Source: BMC Genomics. 2015 Nov 17;16:958. doi: 10.1186/s12864-015-2148-2 (PMC4650926; doi:10.1186/s12864-015-2148-2)

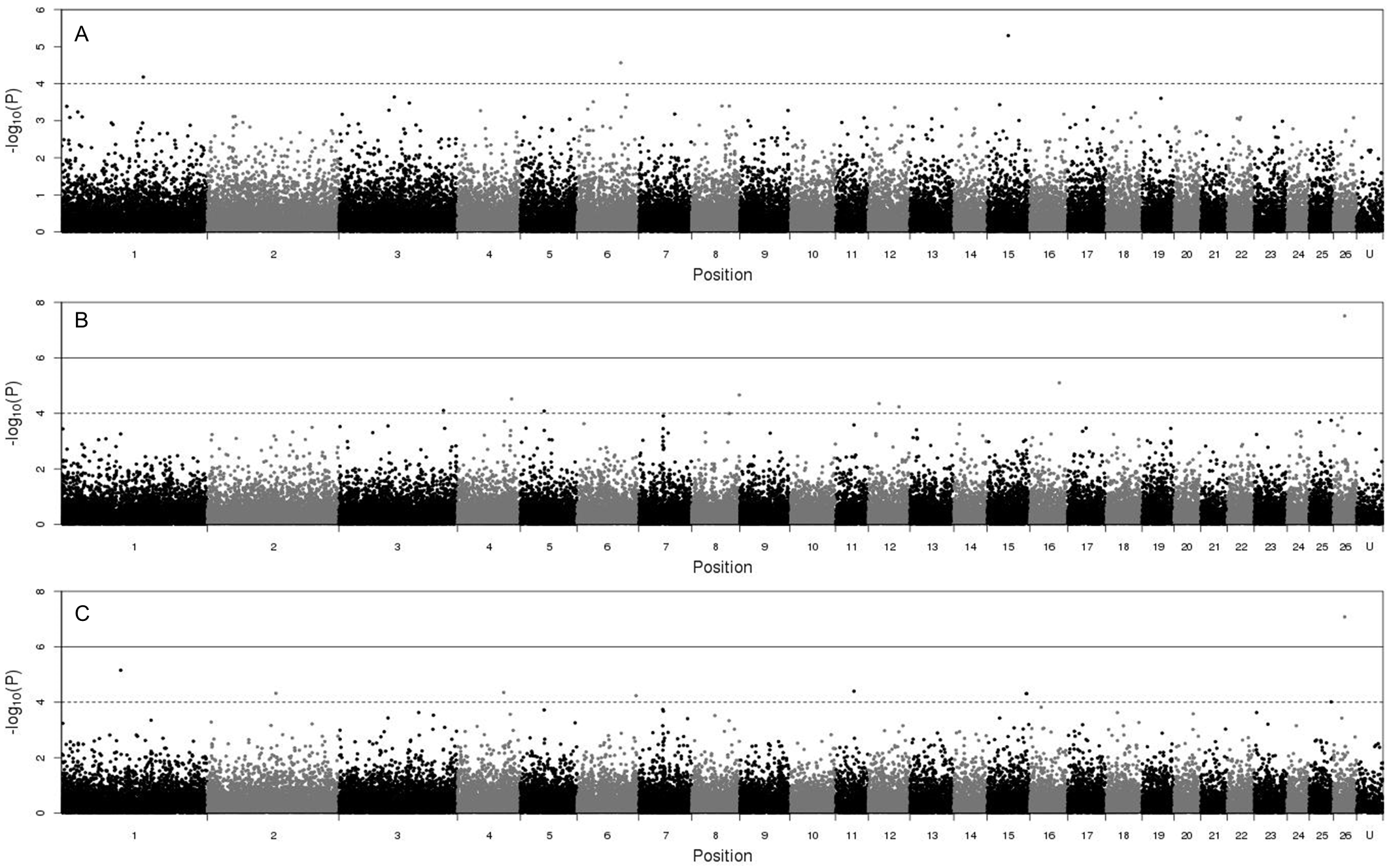

Supplement: Additional file 2: — Manhatten plot of -log 10 ( P ) values of SNPs for dag score at three months (A) and faecal egg count in autumn (B) and as adult (C). Ordered on the ovine genome v3 map, P < 0.0001 (solid line), P < 0.001 (dash line). (PNG 916 kb) [file 12864_2015_2148_MOESM2_ESM.png]
